# Alterations in Ileal Mucosa Bacteria Related to Diet Complexity and Growth Performance in Young Pigs

**DOI:** 10.1371/journal.pone.0108472

**Published:** 2014-09-23

**Authors:** Crystal L. Levesque, Seema Hooda, Kelly S. Swanson, Kees de Lange

**Affiliations:** 1 Department of Animal Science, South Dakota State University, Brookings, South Dakota, United States of America; 2 Department of Animal and Poultry Science, University of Guelph, Guelph, Ontario, Canada; 3 Department of Animal Sciences, University of Illinois-Urbana Champaign, Urbana, Illinois, United States of America; University of Padova, Medical School, Italy

## Abstract

**Background:**

Evaluation of the prolonged impact of weaning diet on ileal mucosa bacteria and during periods of reduced and improved growth was conducted using 454 pyrosequencing.

**Methodology/Principal Findings:**

Weaned pigs were fed HIGH or LOW complexity diets, with or without antibiotics, for 6 weeks, followed by a common grower diet. Pigs were killed at 2 (n = 4 or 5) and 8 (n = 6) weeks post-weaning (periods of reduced and improved growth, respectively). Mucosal bacteria were removed; DNA was extracted and amplified using the V1–V3 region of the 16S rRNA gene. Mucosal bacteria clustered more closely by week post-weaning than diet but 44% of bacterial species did not change from week 2 to 8. There was no effect of diet complexity or antibiotic inclusion on indices of bacterial diversity. Firmicutes made up 91 and 96% of total reads at week 2 and 8, respectively. The proportion of *Clostridium paraputrificum* increased (*P* = 0.003) from week 2 to 8 in pigs fed LOW but didn’t change in pigs fed HIGH; whereas *Clostridium leptum* decreased (*P* = 0.02) from week 2 to 8 in pigs fed LOW but didn’t change in pigs fed HIGH. The proportion of *Sarcina* genus was 3-fold higher in pigs fed A+ compared to A− at week 2 and 5-fold higher at week 8 despite the lack of in-feed antibiotics at that time.

**Conclusions/Significance:**

Shifts in mucosal bacteria populations may be related to dietary induced changes in growth performance during reduced and improved growth but further studies are required to confirm causative relationship. Weaning diet results in species specific prolonged alterations in mucosal bacteria, particularly where high levels of in-feed antibiotics are used. A considerable portion of ileal mucosal bacteria colonize early and remain stable over time despite changes in diet.

## Introduction

The swine industry is under increasing pressure to improve production efficiencies while maintaining overall production [1] and to reduce or eliminate the use of in-feed antibiotics [2]. The starter phase represents a period of the highest diet costs and the greatest inclusion of in-feed antibiotics. Lower cost simple starter diets and removal of in-feed antibiotics can be used to address these challenges but both strategies are associated with reduced nursery performance and increased risk of post-weaning diarrhea [3]. To make informed decisions regarding starter pig feeding programs and to manage concerns for post-weaning health control an understanding of the physiological mechanisms involved in periods of reduced and improved growth (i.e. gut architecture and function, expression of genes related to growth, digestion, and immune function, and gut microbiology) are necessary. In a large scale production trial, simple diets, with or without antibiotics reduced piglet performance in the starter phase but improved growth was observed in the early grower-finisher phase, based on a compensatory growth index such that days to market and carcass characteristics at slaughter were not affected [4]. Overall feed costs were reduced up to $5/pig by using simple or antibiotic-free diets in the nursery phase. However, the capacity to compensate for early dietary challenge was influenced by disease indicating that early dietary challenge may permanently alter physiological mechanisms involved in regulating the capacity to withstand weaning-associated stress and health challenges.

Therefore, assessment of potential mechanisms involved in the observed reduced and improved growth performance of pigs from the large scale trial [4] was conducted. Given the importance of the intestinal microbiome on the health and growth performance of pigs, the impact of early dietary challenge on the intestinal microbiome was evaluated. Denaturing gradient gel electrophoresis was used to obtain a global assessment of changes in the ileal microbial community diversity over time, as influenced by early post-weaning nutrition, prior to more detailed sequencing analysis [5]. The results suggested that post-weaning nutrition had a prolonged effect and permanently altered ileal mucosa-associated, but not ileal digesta, microbiota compositions. Furthermore, differences in diversity of greater than 30% between groups of pigs indicated that further sequencing analysis was warranted. Given the similar bacterial profile (>85% similarity coefficient) in the ileal digesta between groups of pigs in the growing phase [5] further sequencing was conducted on the ileal mucosa-associated microbiome rather than the digesta microbiome. The following study describes the characterization of the short-term and prolonged alterations in ileal mucosa-associated bacteria in pigs following a dietary challenge immediately post-weaning using 16S ribosomal RNA (16S rRNA) gene pyrosequencing. The objective was to 1) describe the ileal mucosa bacterial profile during periods of reduced and improved growth following an early dietary challenge and 2) determine whether early dietary challenge permanently altered the profile of ileal mucosa bacterial populations.

## Materials and Methods

### Ethics statement

All procedures were approved by the University of Guelph Animal Care Committee (AUP 09RO43) and followed the Canadian Council of Animal Care guidelines.

### Animals and diets

The following study was part of a large production trial (i.e. growth performance from weaning to market weight) [4] where five hundred and fifty-two Yorkshire barrows and gilts were weaned at three weeks of age (7·03±0·07 kg BW) in five blocks at the Arkell Swine Research Station (University of Guelph, Guelph, Ontario, Canada). Groups of pigs were fed a high or low complexity nursery diet with (A+) or without (A−) in-feed antibiotics (2.73 g chlortetracycline per kg complete diet, Alpharma, Missisauga, ON, Canada) designated as HIGHA+, HIGHA−, LOWA+, LOWA− in a 3-phase feeding regimen (Table 1). Within each phase, experimental diets were formulated to be similar in energy and amino acid content and were fed from weaning to six weeks post-weaning. The high complex diets contained typical levels of highly digestible, complex animal protein sources (i.e. whey, fishmeal, spray-dried blood meal, and blood plasma) while the low complex diets contained primarily corn and soybean meal with whey and fishmeal included for the first week post-weaning only. A total of forty-eight pigs were selected from three of the five blocks of pigs (two pigs per dietary treatment per block) and euthanized at week 2 (six pigs per dietary treatment) and week 8 (six pigs per dietary treatment) post-weaning. Pigs selected at week 8 had received a common, non-limiting grower diet from six to eight weeks post-weaning. Nursery and grower diets were fed as crumble and pellets, respectively. Pigs had ad libitum access to feed and water throughout the experiment. To assess possible carry-over effects of weaning phase diet regimen a ‘washout’ period was necessary. The first 2 weeks of the grower phase was selected as the washout period which also coincided with the period of compensatory growth reported by Skinner et al [4].

**Table 1:** Ingredient composition of High and Low complexity diets fed to weaned pigs for six weeks post-weaning and common grower/finisher diets (as-fed basis)^1^.

	Nursery							
	High complexity			Low complexity				
**Ingredient, %**	Phase I	Phase II	Phase III	Phase I	Phase II	Phase III	Grower	
Corn	18·9	38·7	50·2	47·1	49·6	47·3	45·8	
Soybean meal	10·8	15·0	21·0	24·0	34·0	37·0	28·2	
Wheat				10·0	10·0	10·0	20·0	
Barley	25·0	25·0	20·0					
Whey	20·0	8·00		8·00				
Fat, animalvegetable	2·50	2·50	2·50	2·50	2·50	2·50	2·00	
Herring meal	5·00	3·00		5·00				
AP920Blood plasma^2^	4·50	2·00						
Blood meal,spray dried^3^		2·00	2·00					
Oat groats	10·0							
L-Lysine·HCl	0·30	0·25	0·35	0·16	0·25	0·05	0·23	
DL-Methionine	0·18	0·18	0·18	0·06	0·11		0·08	
L-Threonine	0·10	0·12	0·16	0·04	0·09		0·10	
L-Tryptophan	0·02	0·02	0·02					
Limestone	0·50	0·58	0·86	1·00	1·18	1·10	1·22	
Salt		0·20	0·30	0·20	0·30	0·30	0·40	
Monocalcium phosphate	0·80	1·00	1·35	1·30	1·40	1·20	1·42	
Calcium formate	0·40	0·40	0·20					
Calcium propionate	0·40	0·40	0·20					
Saccharine	0·05	0·05	0·05					
Vitamin andmineral mix^4^	0·60	0·60	0·60	0·60	0·60	0·60	0·60	
Calculated composition^5^								
DE, MJ/kg	14·4	14·3	14·5	14·9	14·9	15·0	14·7	
CP, %	20·5	19·8	18·7	21·1	21·8	22·7	19·8	
Lys, %	1·51	1·39	1·29	1·37	1·39	1·30	1·23	
Ca, %	0·85	0·80	0·74	0·85	0·80	0·75	0·80	
Available P, %	0·70	0·65	0·65	0·75	0·70	0·67	0·69	
Na, %	0·37	0·26	0·15	0·20	0·14	0·14	0·18	
Cl, %	0·55	0·43	0·34	0·37	0·29	0·24	0·36	

1 Dietary treatments were diet complexity (High and Low) with or without antibiotic inclusion (Chloratetracycline, 273 g per kg complete feed [added in the form of Aureomycin 220 G], added at the expense of corn) fed from weaning (21 d of age) to 63 d of age (i.e. nursery). All pigs received common commercial grower and finisher diets thereafter.

2 Manufactured by APC Nutrition Inc. (Ames, IA).

3 Manufactured by Rothsay (Guelph, ON, Canada).

4 Vitamin and mineral mix (DSM Nutritional Products Canada Inc., Ayr, ON, Canada) supplied per kg of complete diet: retinol, 4,128 µg; cholecalciferol, 30 µg; D,L-α-tocopherol acetate, 52·8 mg; menadione, 3 mg; vitamin B_12_, 0.03 mg; pantothenic acid, 18 mg; riboflavin, 6 mg; choline, 600 mg; folic acid, 2·4 mg; niacin, 30 mg; thiamine, 18 mg; pyridoxine, 1.8 mg; Cu, 18 mg as CuSO_4_·5H_2_O; Fe, 120 mg as FeSO_4_; Mn, 24 mg as MnSO_4_; Zn, 126 mg as ZnO; Se, 0·36 mg as FeSeO_3_; I, 0·6 mg as KI.

5 Calculated based on NRC (1998) ingredient values.

### Tissue collection, DNA extraction, pyrosequencing, and bioinformatics

A 40-cm section of ileum beginning at a point 20 cm proximal to the ileo-cecal junction was dissected and immediately placed on ice until processing. Bacteria attached to the ileal mucosal wall, termed mucosa bacteria, were removed from the ileal tissue as described previously [6] with minor modifications. Briefly, ileal tissue was opened longitudinally, cut in half lengthwise, and briefly washed three times in saline by gentle agitation to remove unattached or loosely attached bacteria from the wall. Bacterial cells were released from the ileal wall in three 20 ml saline washes with 0.1% (w/w) Tween 80 using a 50-ml conical tube and vigorously shaking the tube for 1 min. The washes were pooled and centrifuged (27,000×**g**) at 4°C for 30 min to pellet the cells. Each half was processed in each of the 20 ml washes for 1-min prior to pooling. Mucosa-associated pellets were stored at −80°C until DNA extraction. Mucosa bacterial DNA extraction was completed using PowerSoil DNA isolation kit (MoBio Laboratories) according to manufacturer’s specifications and DNA was quantified using a NanoDrop ND-1000 spectrophotometer (Nano-Drop Technologies, Wilmington, DE). Genomic DNA quality was assessed by electrophoresis using 1% (w/v) agarose gel with 0.004% (v/v) ethidium bromide. Amplicons were generated using the V1–V3 region of 16S rRNA gene. The forward primer consisted of the sequences of illumina adapter FP1, index N5-series, sequencing primer and a universal 16S V1 primer (5′ AATGATACGGCGACCACCGAGATCTACACTAGATCGCTCGTCGGCAGCGTCAGAGTTTGATCCTGGCTCAG). The reverse primers contained the sequences of illumine adaptor FP2, index N7-series and a universal 16S V3 reverse primer (5′ CAAGCAGAAGACGGCATACGAGATTCGCCTTAGTCTCGTGGGCTCGGATTACCGCGGCTGCTGGC). The PCR reaction mix (25 µL) contained 1× PCR buffer, 200 µM each of dNTP, 0.8 µM of each primer pair, and 2.5 U of HotStarTaq DNA Polymerase (Qiaqen). The PCR thermal cycling conditions were 95°C for 15 min, 30 cycles of 94°C for 15 sec, 55°C for 45 sec and 72°C for 40 sec, followed by 72°C for 8 min using a GeneAmp PCR System 9700 thermal cycler. After PCR, amplicons were purified from the agarose gel to remove any smaller fragment or primer-dimer using QIAGEN gel purification kit. Further DNA concentration and quality were measured using a NanoDrop 2000 spectrophotometer and Aligent 2100 BioAnalyzer with a high sensitivity DNA chip electrophoresis, respectively.

The PCR amplicons were combined in equimolar ratios to create a DNA pool (20 ng in 1 µl) that was used for pyrosequencing. Pyrosequencing of the PCR amplicons was performed at the W. M. Keck Center for Biotechnology at the University of Illinois using a 454 Genome Sequencing and FLX titanium reagents (Roche Applied Science, Indianapolis, IN). After sequencing was completed, all reads were scored for quality and any poor quality reads and primer dimers were removed. High quality (quality value >25) sequence data derived from the sequencing process was processed using a proprietary analysis pipeline (www.mrdnalab.com) and as described previously [7]–[13]. Briefly, sequences were depleted of barcodes and primers, short sequences (<200 bp), sequences with ambiguous base calls, and sequences with homopolymer runs exceeding 6 bp. Sequences were then denoised and chimeras were removed. Operational taxonomic units (OTU) were defined after removal of singleton sequences and clustering at 3% divergence (97% similarity). OTUs were then taxonomically classified using BLASTn against a curated GreenGenes database [14] and compiled into each taxonomic level into both “counts” and “percentage” files. All raw sequence data will be available at the NCBI sequence read archive (http://www.ncbi.nlm.nih.gov/Traces/sra/) under accession numbers SAMN03003724 to SAMN03003767.

### Statistics

Relatedness of samples was tested and represented by creating a dual hierarchical clustering dendogram and performing principal component analysis (PCA) as described by Cephas et al [15]. Data presented as percentage of sequences at each taxonomic level were analyzed as a repeated measures in a 2×2 factorial treatment design using the PROC MIXED procedure of SAS (version 9.2, SAS Institute) using pig as the experimental unit. The model included the main effects of diet complexity, antibiotic inclusion, week post-weaning, and their interaction. Pig nested within block and diet was the random variable. Means were separated using Fisher-protected least significant difference with Tukey adjustment. Where the proportion of a given bacterial species changed from week 2 to week 8, Pearson correlation coefficients were determined between pig performance at each week and the identified species. Results are reported as least squares means ± standard error where p≤0.05 and 0.05<p≤0.10 was considered significant and trend, respectively.

## Results

Performance of all pigs in the large scale trial has been previously published [4]. In that paper we reported that pigs fed low complex (i.e. Simple) or antibiotic-free diets had reduced growth in the nursery phase but demonstrated improved growth in the early grower-finisher phase compared to pigs fed high complexity diets with in-feed antibiotics. Pigs fed the low complex without antibiotic diet (i.e. Simple A−) in the nursery demonstrated the largest improvement in growth in the early grower-finisher phase and there was no impact of diet complexity or antibiotic inclusion on market weight, days to market, or carcass characteristics at slaughter. Growth performance of the pigs selected for collection of digesta and ileal mucosal bacteria for the current study are summarized in Table 2 and show a similar trend to that reported in the large scale trial [4]. Daily gain in the first 2 weeks post-weaning was reduced in pigs (*P* = 0.05) fed LOW diets but there was no difference in gain between groups at week 8 post-weaning. Body weight showed a similar response to diet complexity and antibiotic inclusion. There was no difference in feed intake between treatment groups at week 2 or 8 post-weaning (data not shown).

**Table 2:** Pig performance at week 2 and 8 post-weaning in pigs fed diets differing in diet complexity and with or without antibiotic inclusion^1^.

Diet complexity	HIGH		LOW			*P* 2			
	A−	A+	A−	A+	SEM	Comp	A	Comp× A	
Week 2 (35 d of age) post-weaning									
Body weight, kg	9.92	10.4	9.63	9.48	0.29	0.05	0.56	0.28	
Average daily gain, g/d	322	352	260	278	33	0.05	0.48	0.87	
Week 8 (77 d of age) post-weaning									
Body weight, kg	38.4	40.6	39.0	43.1	1.8	0.40	0.11	0.60	
Average daily gain, g/d	820	848	835	832	45	0.99	0.78	0.74	

1 Dietary treatments were diet complexity (High and Low) with or without antibiotic inclusion (Chloratetracycline, 273 g per kg complete feed [added in the form of Aureomycin 220 G], added at the expense of corn) fed from weaning (21 d of age) to 63 d of age (i.e. nursery). All pigs received common commercial grower and finisher diets thereafter. Means values with their pooled standard errors.

2 A, antibiotic inclusion; Comp, diet complexity.

**Figure 1:**
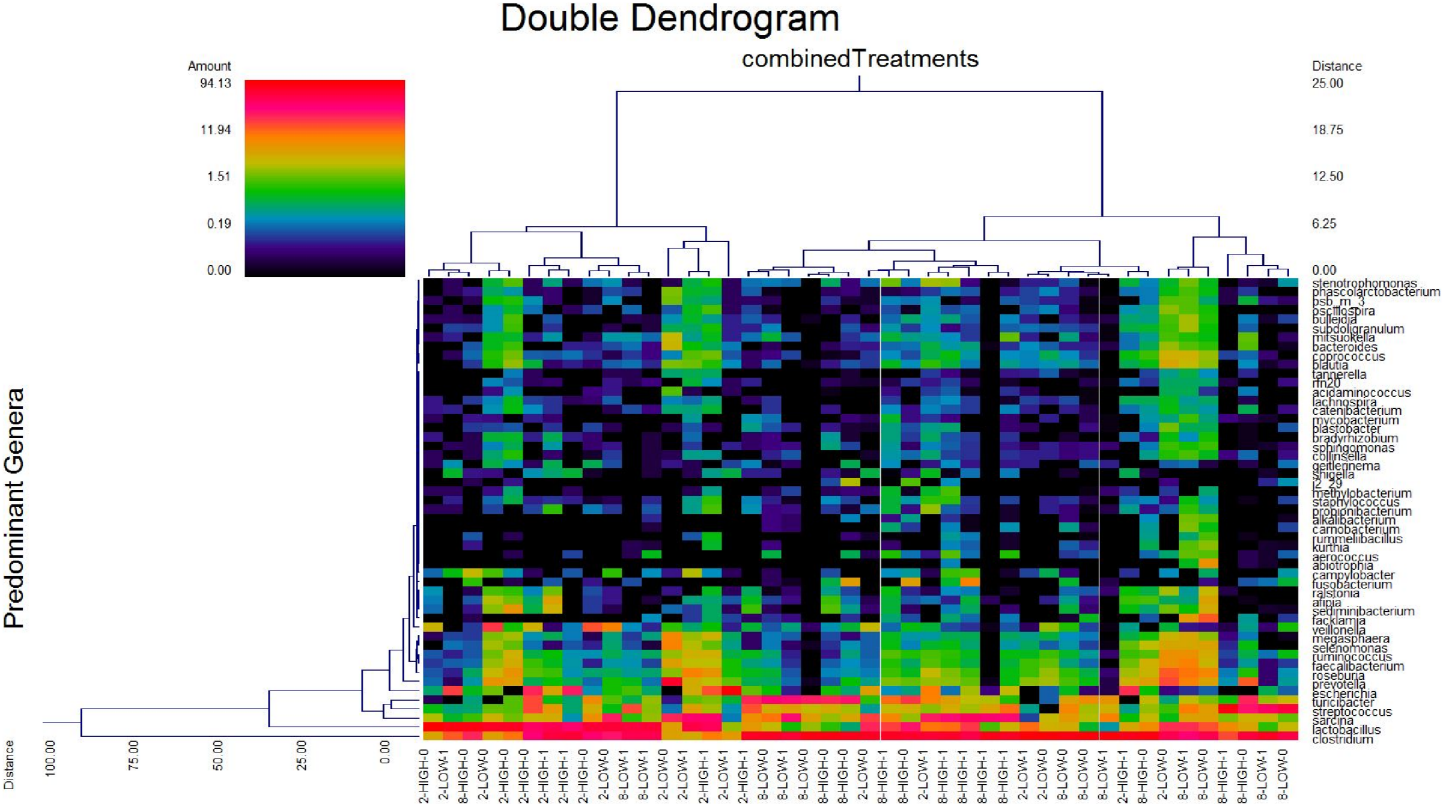
Dual hierarchical clustering dendogram of the fifty most abundant bacterial genera among the pig samples collected at 2 and 8 weeks post-weaning and fed HIGH or LOW complex diets with (1) or without (0) in-feed antibiotic. Experimental diets were fed from 0 to 6 weeks post-weaning. All pigs received the same grower/finisher diets thereafter. The double dendrogram is based on the Wards clustering and Manhattan distance methods. The heat map depicts the relative percentage of each genus for each sample. The relative distance scale for the left y-axis is provided in the lower left corner of the figure. The colour scale for the heat map is shown in the upper left corner of the figure.

**Figure 2:**
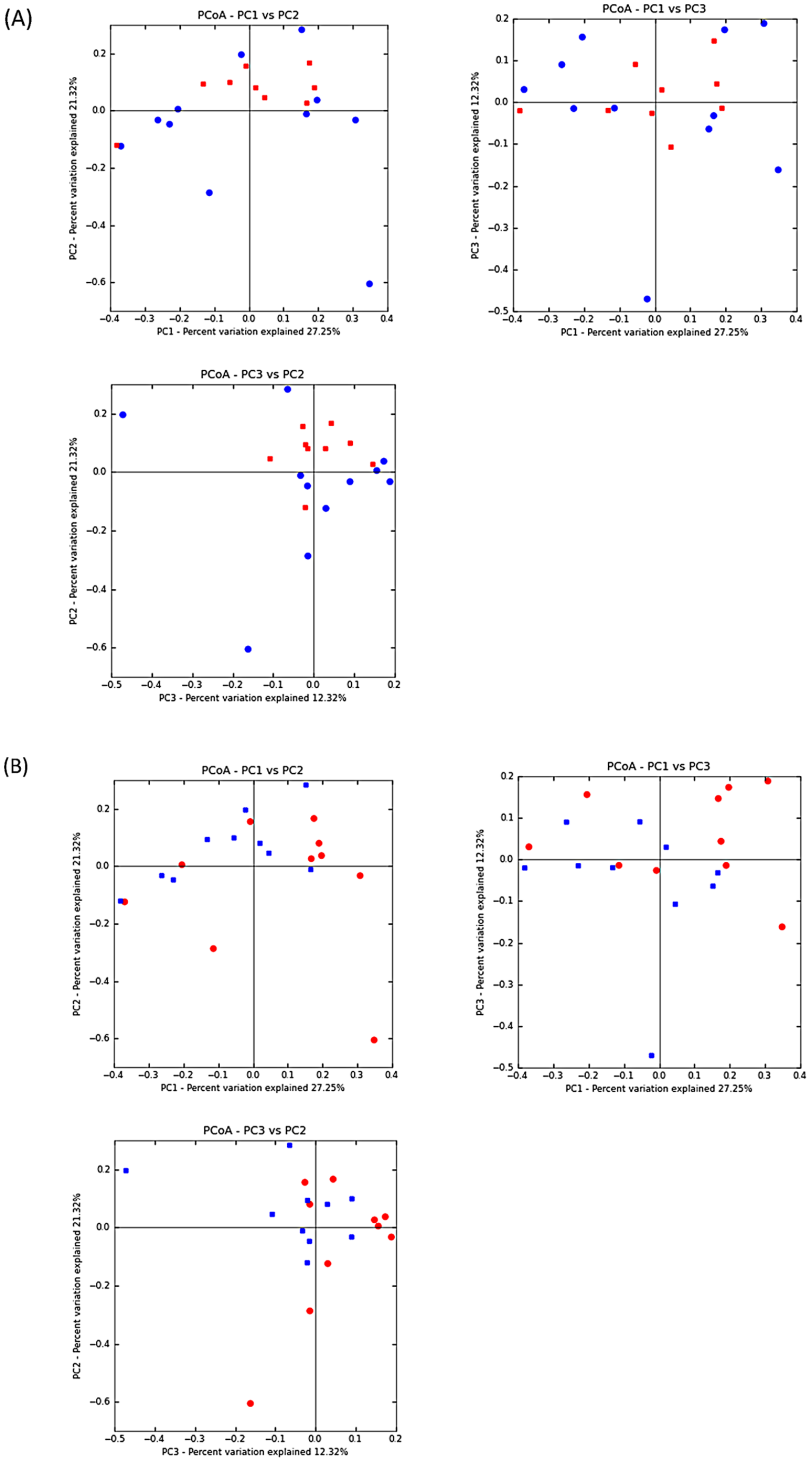
Principal component analysis of Uni Frac distance metric. Plot (A) was generated using sequences from individual pigs at week 2 post-weaning fed High (blue circles) or Low (red squares) complexity diets from 0–6 weeks post-weaning. Plot (B) was generated using sequences from individual pigs at week 2 fed diets with (blue squares) or without (red circles) antibiotics. All pigs received a common grower diet thereafter.

**Figure 3:**
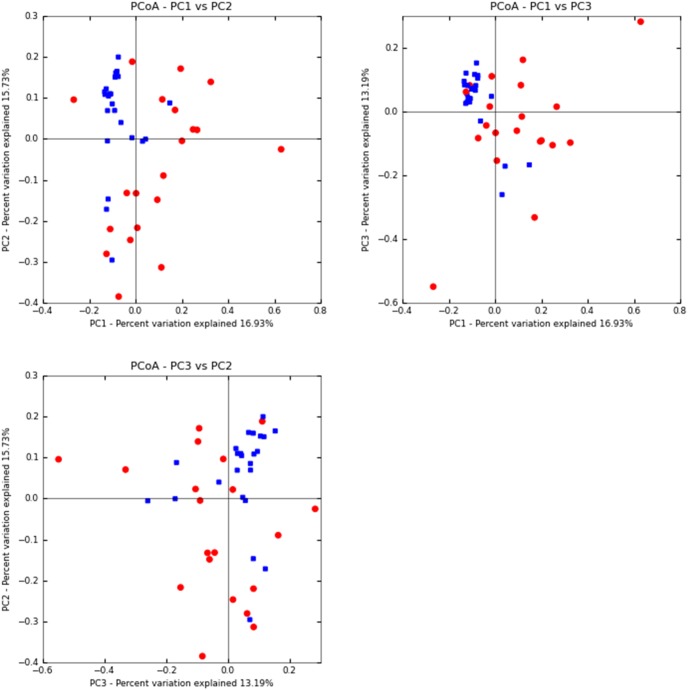
Principal component analysis of Uni Frac distance metric. The plot was generated using sequences from individual pigs at week 2 (red circles) and 8 (blue squares) post-weaning fed diets differing in diet complexity and with or without antibiotic inclusion from week 0–6 post-weaning. All pigs received a common grower diet thereafter.

At week 2 post-weaning, a total of eighteen pigs (n = 4, 5, 5, and 4 for HIGHA−, HIGHA+, LOWA−, and LOWA+, respectively) were used for microbial analysis. Six pigs were removed due to low DNA quality. At week 8 post-weaning, high quality DNA was obtained for all pigs (n = 6 for all treatment groups). Pyrosequencing of 16S rRNA gene barcoded amplicons resulted in a total of 311,245 and 349,524 high-quality sequences (mean of 12,780 and 14,437 sequences per sample) at week 2 and 8, respectively. The observed OTU and Shannon index were not different (*P*>0.10) among the treatment groups within week post-weaning (data not shown) indicating that mucosa bacterial diversity was not different among dietary treatments. However, microbial diversity tended to be greater (*P*<0.10) at week 8 than week 2 post-weaning based on Shannon index (5.05 vs. 4.40, week 8 and 2 post-weaning, respectively) and observed OTU (605 vs. 495, week 8 and 2 post-weaning, respectively). The dual hierarchical clustering dendogram of the fifty most abundant bacterial genera indicated a clustering of samples based on week post-weaning where *Lactobacillus* were more abundant at week 2 and *Clostridium* were more abundant at week 8 post-weaning (Fig. 1). Regardless of week post-weaning *Clostridium, Lactobacillus, Sarcina,* and *Streptococcus* were the predominant genera. Further analysis using UniFrac PCA (Fig. 2A) showed that at week 2 post-weaning, pigs fed HIGH appeared to cluster more closely than pigs fed the LOW and pigs fed A+ appeared to cluster more closely than pigs fed A− (Fig. 2B). There was no segregation between treatment groups at week 8 post-weaning (data not shown). However, pigs at week 8 clustered separately from pigs at week 2 post-weaning (Fig. 3).

A total of sixteen phyla, twenty-eight classes, fifty-three orders, 110 families, 236 genera, and 403 species were annotated but within each taxonomical level only those with ≥1% of the reads were considered for analysis. The primary phyla at week 2 post-weaning were Firmicutes, Proteobacteria, and Bacteroidetes at 91.5, 5.6, and 2.0% of total sequences, respectively, and were not influenced by diet complexity or antibiotic inclusion. Diet complexity had no effect on the microbial population at the level of class or order. Among the Firmicutes, the proportion of the Clostridia class was higher (*P* = 0.06) at week 8 than week 2 post-weaning, being dominated by the Clostridiales order (67.2 vs 51.5±5.9% of total sequences). There was no difference between week post-weaning for the Bacilli class (35.1±8.2% of total sequences). However, the proportion of the Turicibacterales order was higher (*P* = 0.04; 6.4 vs 2.0±1.5% of total sequences, respectively); whereas the proportion of the Lactobacillales order tended to be lower (*P* = 0.08; 22.3 vs 37.6±6.4% of total sequences, respectively) at week 8 than week 2 post-weaning. Among the Proteobacteria, the Gammaproteobacteria class was lower (*P*<0.001) at week 8 than week 2 post-weaning, being dominated by the Enterobacterales order (4.5 and 0.3±0.9% of total sequences at week 2 and 8 post-weaning, respectively). There were minor effects of diet complexity at the level of bacterial family. At week 2 post-weaning pigs fed HIGH diets had lower (*P* = 0.01) proportions of the family Veillonellaceae than pigs fed LOW diets (1.6 and 5.9±1.5% of total sequences, respectively). The inclusion of antibiotics tended to increase (*P* = 0.09) the proportion of the Clostridiaceae family at week 2 post-weaning (32.2 and 14.7±6.1% of total sequences in A+ and A− fed pigs, respectively). There was no effect of diet complexity on the proportion of bacterial genera (Table 3) or species (Table S1). Within the Firmicutes phyla, the proportion of the *Veillonella* was greater (*P* = 0.05) in pigs fed A− than A+ diets (5.48 and 0.51±1.57% of total sequences, respectively). Conversely, the proportion of the *Escherichia* (*P* = 0.08) genus tended to be lower in pigs fed A− diets (2.36 and 6.51±1.89% of total sequences, respectively). The proportion of *Sarcina* was also 3-fold higher in pigs fed A+ compared to A−. There tended to be an interaction (*P* = 0.07) between diet complexity and antibiotic inclusion; where, the proportion of sequences associated with *Veillonella* were greater (*P*<0.01) in pigs fed LOWA− than LOWA+ but not different in pigs fed HIGHA− and HIGHA+. Pigs fed A− has higher (*P* = 0.01) proportions of *Lactobacillus salivarus* than pigs fed A+ and the difference was greater (*P* = <0.05) in HIGH than LOW fed pigs (Table S1).

**Table 3:** Mucosa bacterial genera (expressed as a percentage of sequences) on the ileal mucosa of pigs fed diets differing in diet complexity or antibiotic inclusion at week 2 and 8 post-weaning based on 16S rRNA gene sequencing^1^.

Diet complexity	HIGH		LOW		*P* 2						
Genus	A−	A+	A−	A+	SEM	Comp	A	Comp×A			
Week 2 (35 d of age) post-weaning											
Firmicutes	92.6	88.5	93.0	92.7	3.4	0·51	0·49	0·55			
*Lactobacillus*	61.4	21.9	30.6	34.1	13.6	0•51	0•23	0•17			
*Clostridium*	15.5	49.0	43.9	38.9	15.6	0•57	0•39	0•26			
*Sarcina*	2.31	5.69	4.19	13.2	5.71	0•43	0•28	0•61			
*Streptococcus*	3.34	2.62	0.29	0.94	1.54	0•18	0•98	0•66			
*Turicibacter*	3.63	3.19	0.23	1.33	2.09	0•25	0•88	0•72			
*Veillonella*	1.42^b^	0.94^b^	9.53^a^	0.08^b^	2.61	0•21	0•05	0•07			
*Ruminococcus*	0.56	0.46	1.12	0.71	0.57	0•49	0•65	0•78			
*Faecalbacterium*	0.66	0.34	1.20	0.68	0.54	0•43	0•44	0•84			
*Lachnospira*	0.08	0.06	0.08	0.03	0.05	0•81	0•44	0•81			
Bacteroidetes	1.83	1.10	4.10	1.36	1.65	0·47	0·32	0·56			
*Prevotella*	0.76	0.58	3.32	1.05	1.26	0•25	0•34	0•41			
Proteobacteria	4.90	9.98	2.49	5.77	2.63	0·23	0·15	0·73			
*Escherichia*	3.94	8.44	0.78	1.58	2.82	0•26	0•08	0•90			
Week 8 (77 d of age) post-weaning											
Firmicutes	94.9	69.4	96.8	96.8	1.8	0·53	0·69	0·71			
*Clostridium*	59.0	52.4	66.3	52.4	9.3	0•70	0•30	0•70			
*Lactobacillus*	17.6	6.1	2.5	19.7	8.4	0•93	0•74	0•13			
*Streptococcus*	4.15	16.5	10.3	8.74	8.41	0•93	0•54	0•43			
*Sarcina*	1.39^a^	17.0^b^	2.75^a^	5.04^a^	2.90	0•10	0•01	0•05			
*Turicibacter*	10.4	2.10	8.73	4.56	3.15	0•90	0•08	0•53			
*Ruminococcus*	0.22	0.26	0.46	0.66	0.34	0•38	0•74	0•81			
*Faecalbacterium*	0.35	0.19	0.32	0.86	0.46	0•51	0•69	0•47			
*Veillonella*	0.11	0.02	0.14	0.04	0.04	0•62	0•07	0•63			
*Facklamia*	0.03	0.06	1.42	0.50	0.72	0•24	0•55	0•53			
*Lachnospira*	0.03	0.02	0.04	0.02	0.02	0•89	0•47	0•88			
Bacteroidetes	2.36	0.61	1.12	1.20	0.99	0·75	0·43	0·38			
*Prevotella*	2.04	0.04	0.57	0.92	0.82	0•56	0•47	0.27			
Proteobacteria	1.10	1.71	1.39	0.98	0.61	0·77	0·90	0·42			
*Escherichia*	0.16	0.45	0.10	0.15	0.15	0•26	0•28	0.43			

1 Bacterial genera with sequences of <1% of total reads were not included for statistical analysis. Pigs were fed diets differing in diet complexity and antibiotic inclusion from weaning (21 d of age) to 63 d of age (week 6 post-weaning). All pigs received the same grower-finisher diets from 64 to 78 d of age. At week 2 post-weaning *n* = 4, 5, 5, and 4 for HighA−, HighA+, LowA−, and LowA+, respectively. At week 8 post-weaning *n* = 6 for all treatment groups. Means values with their pooled standard errors.

2 A, antibiotic inclusion; Comp, diet complexity. Within main effect of diet complexity or antibiotic inclusion, means within a row without common superscript differ ^a,b^ *P*<0.05 (Tukey’s means separation test).

At week 8 post-weaning, the 3 primary phyla were the same as week 2; although, the proportion of Firmicutes was greater (96.1 vs 91.5% of total sequences, *P*<0.01) and the proportion of Proteobacteria was smaller (1.3 vs 5.6% of total sequences, (*P*<0.01) at week 8 compared to week 2. The increase in Firmicutes at week 8 was due primarily to an increase (*P*<0.05) in *Clostridium*, *Streptococcus*, and *Turicibacter*, while *Lactobacillus* and *Escherichia* decreased (*P*<0.01) from week 2 to week 8. The genus *Facklamia* was present at week 8 but not week 2 (Table 3). At week 8, diet complexity had an effect on the proportion of *Sarcina* only, where pigs fed LOW in the starter phase tended to have lower (*P* = 0.10) proportions of *Sarcina* than pigs fed HIGH (3.90 and 9.14±2.05% of total sequences, respectively). Within week 8, pigs fed A− in the starter phase had higher levels of *Turicibacter* (*P* = 0.08; 9.56 and 3.33±2.23% of total sequences, respectively) and *Veillonella* (*P* = 0.07; 0.12 and 0.03±0.03% of total sequences, respectively) but lower *Sarcina* (*P* = 0.01; 2.02 and 11.02±2.05% of total sequences, respectively) than pigs fed A+. As well, there was an interaction between diet complexity and antibiotic inclusion for *Sarcina*, where pigs fed HIGHA+ in the starter phase had higher (*P* = 0.02) *Sarcina* levels than pigs fed HIGHA− but there was no difference between pigs fed LOWA+ and LOWA−.

**Figure 4:**
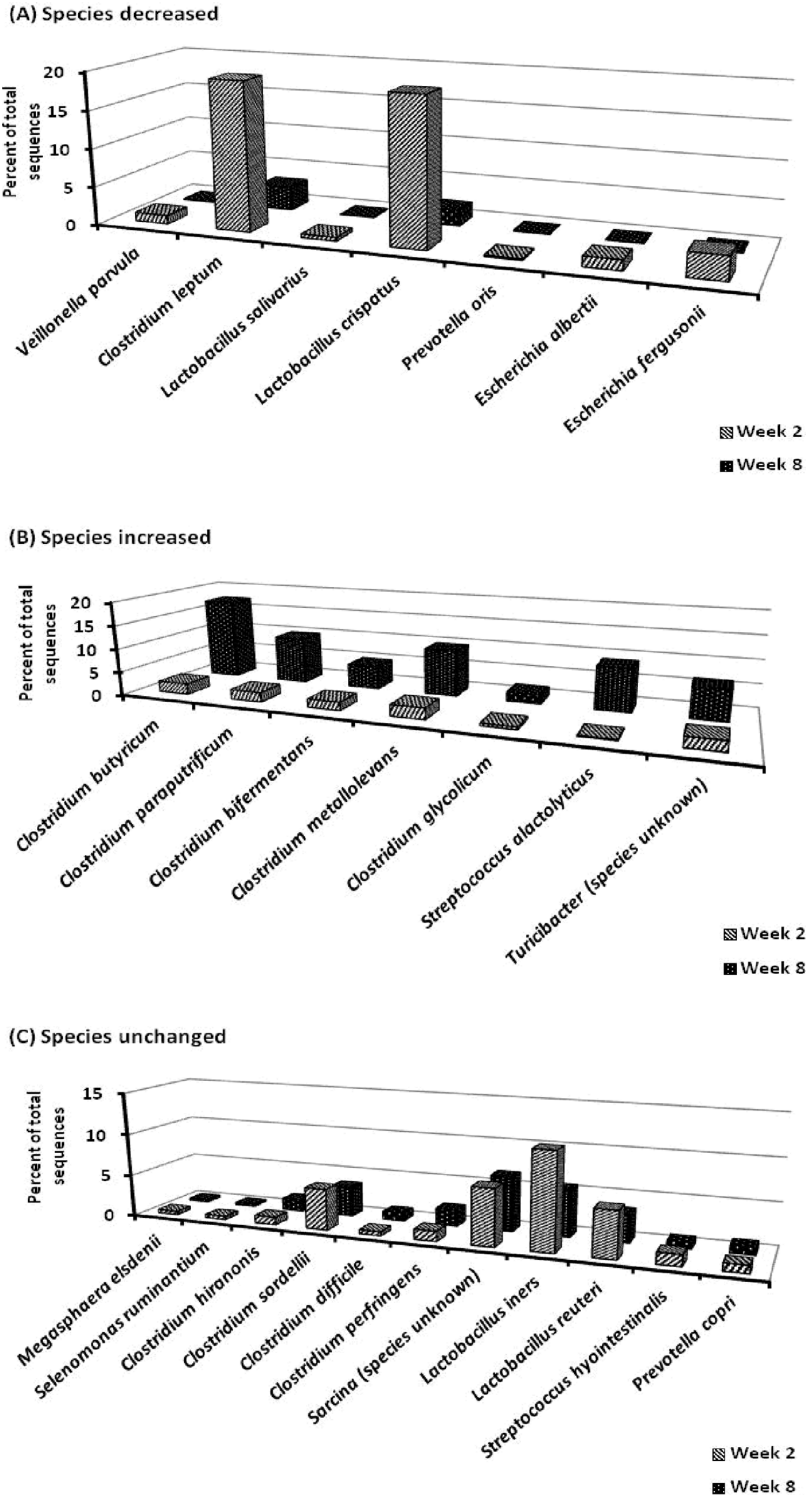
Ileal mucosa bacterial species (expressed as percentage of sequences) in pigs from week 2 to 8 post-weaning. Of the 25 bacterial species with levels of sequencing ≥1% of total sequences seven decreased (A), seven increased (B), and eleven remained unchanged (C) from week 2 to 8 post-weaning. Values are least squared means.

A total of twenty-five out of the 403 annotated species were present at levels of at least 1% of total sequences (Fig. 4); seven increased (*Veillonella parvula, Clostridium leptum, Lactobacillus salivarius, Lactobacillus crispatus, Prevotella oris, Escherichia albertii,* and *Escherichia fergusonii*; Fig. 4A), seven decreased [*Clostridium butyricum, Clostridium paraputrificum, Clostridium bifermentas, Clostridium metallolevans, Clostridium glycolicum, Streptococcus alactolyticus,* and Turicibacter (species unknown) Fig. 4B], and eleven remained unchanged with week post-weaning (Fig. 4C). For both week 2 and 8 post-weaning, the unchanged species made up approximately 20% of the total sequences and 64% of the unchanged species were from the order Clostridiales.

There was a week post-weaning x diet complexity and week post-weaning x antibiotic inclusion interaction for four and five bacterial species, respectively and six of the species made up less than 3% of the total sequences each. The remaining three were from the *Clostridium* genus (*Clostridium paraputrificum, Clostridium leptum, and Clostridium bifermentans*; Table 4). *Clostridium paraputrificum* increased (*P* = 0.003) from week 2 to 8 post-weaning in pigs fed LOW but was not different in pigs fed HIGH. *Escherichia albertii* and *Lactobacillus salivarius* decreased (*P*<0.05) from week 2 to 8 post-weaning in pigs fed HIGH but were not different in pigs fed LOW. The proportion of *Clostridium leptum* decreased (*P* = 0.02) from week 2 to 8 in pigs fed LOW but didn’t change in pigs fed HIGH and were not different between diet complexity at week 8. *Veillonella parvula* and *Lactobacillus salivarius* tended to decrease (*P* = 0.08) from week 2 to 8 in pigs fed A− but was not different between week post-weaning in pigs fed A+. *Escherichia fergusonii* was higher (*P* = 0.02) in pigs fed A+ than A− at week 2 but was not different at week 8. *Clostridium difficile* and *Clostridium bifermentans* decreased (*P*<0.05) from week 2 to 8 in pigs fed A− but was not different between week post-weaning in pigs fed A+. There was a trend (*P* = 0.08) for a week post-weaning x diet complexity interaction for *Sarcina* species (4.41 vs. 9.03±2.88% and 9.14 vs. 3.89±2.46% in pigs fed HIGH vs LOW at week 2 and 8, respectively) but the Tukey’s means comparison was not significant. Proportions of *Clostridium paraputrificum, Clostridium leptum,* and *Sarcina* averaged at least 5% of the reads at week 2 and 8 thus were used for correlation analysis. The proportion of *Clostridium leptum,* and *Sarcina* were negatively correlated with pig performance at week 2 but the correlation coefficients were low (i.e. r<0.25). There was a positive correlation (r = 0.57; *P*<0.01) between the proportion of *Clostridium paraputrificum* and pig body weight at week 2 (Table S2). The proportion of *Clostridium paraputrificum* and *Sarcina* were negatively correlated with pig performance at week 8 but the correlation coefficients were low (i.e. r<0.25). The proportion of *Clostridium leptum* was a positively correlated (r = 0.40; *P* = 0.05) with average daily gain at week 8.

**Table 4:** Mucosa bacterial species (expressed as percentage of sequences) on the ileal mucosa of pigs fed diets differing in complexity or antibiotic inclusion at week 2 and 8 post-weaning with a significant interaction between week post-weaning and diet complexity or antibiotic inclusion based on 16S rRNA sequencing^1^.

	Diet complexity				
	HIGH		LOW		
Species	Week 2	Week 8	Week 2	Week 8	SEM
*Clostridium paraputrificum*	3•24^a^	6•51^a,b^	0•77^a^	13•3^b^	2•42
*Escherichia albertii*	2•24^a,x^	0•09^b^	0•42^b,y^	0•05^b^	0•48
*Lactobacillus salivarius*	0•91^a^	0•05^b^	0•05^b^	0•03^b^	0•13
*Clostridium leptum*	11•6^a,x^	5•3^x^	27•2^a,y^	1•03^b^	6•41
	**Antibiotic Inclusion**				
	**A−**		**A+**		
	**Week 2**	**Week 8**	**Week 2**	**Week 8**	
*Veillonella parvula*	2•12^x^	0•08^y^	0•09^y^	0•03^x,y^	0•61
*Clostridium difficile*	0•14^x^	1•35^y^	0•78^x,y^	0•54^x,y^	0•38
*Clostridium bifermentans*	0•67^a^	6•84^b^	2•76^a,b^	3•15^a,b^	1•62
*Lactobacillus salivarius*	0•95^a^	0•04^b^	0•01^b^	0•03^b^	0•13
*Escherichia fergusonii*	1•24^a^	0•11^a^	4•93^b^	0•18^a^	0•83

1 Pigs were fed diets differing in diet complexity and antibiotic inclusion from weaning (21 d of age) to 63 d of age (week 6 post-weaning). All pigs received the same grower-finisher diets from 64 d of age to market weight (110 kg). At week 2 post-weaning *n* = 12, 10, 11, and 11 for High, Low, A−, and A+. At week 8 post-weaning *n* = 12 for all treatment groups. Values are least squares means with their pooled standard errors. A, antibiotic inclusion. Within diet complexity or antibiotic inclusion, means within a row without common superscript differ ^a,b^ *P*<0.05, ^x,y^ *P*<0.10 (Tukey’s means separation test).

## Discussion

The importance of the intestinal microbiome on the growth and performance of the host as a result of microbial influences on nutritional, physiological, and immunological processes are well established [16]. With the use of high-throughput DNA sequencing, detailed descriptions of the composition and diversity of the microbiome are possible [17] and can be used to gain a better understanding of the important metabolic contributions of specific microbial populations to host growth and performance or response to disease.

The use of culture-independent DNA sequencing continues to increase, but the extent of knowledge about the bacterial microbiome of the pigs is still limited [18], thus it is important to first evaluate whether the general bacterial profiles, based on next generation sequencing, observed in the current study are consistent with those recently reported. The clustering of mucosa bacterial profiles by week post-weaning in the current study was supported by our initial assessment based on denaturing gradient gel electrophoresis [5]. As well, the fecal bacterial profile in two commercial herds separated more strongly by age than herd [18]. In our initial assessment, the mucosa bacteria further clustered by antibiotic inclusion within week 2, thus the lack of clustering or difference in diversity based on antibiotic inclusion at week 2 was unexpected. However, Rettedal et al [19] also reported no distinct clustering or differences in diversity (based on Shannon index) in pigs fed with or without in-feed chlortetracycline at 50 ppm in the initial 2 weeks post-weaning. Firmicutes and Proteobacteria made up >97% of the ileal mucosa bacteria phyla and are supported by Isaacson and Kim [17] who report Firmicutes and Proteobacteria as being the 2 dominant phyla in the ileum, with Proteobacteria making up 5–40%. At the genera level, *Clostridium*, *Lactobacillus* and *Sarcina* made up 81% of the total sequences, which is similar to that reported for the ileal digesta and mucosa of pigs at 2 weeks post-weaning where the 3 primary genera were *Lactobacillus*, *Turicibacter*, and members of the order Clostridiales [19].

Changes in the composition of the intestinal microbiotia over time have been reported for ileal digesta in neonatal, unweaned and weaned pigs [20], fecal microbiota during the grower-finisher phase [18], [21] or from birth to 30 d of age [22] but to the authors’ knowledge, this is the first study to examine changes in ileal mucosa bacterial profiles from the weaning to the growing phase. The increase in the proportion of bacteria of the Firmicutes phyla with age observed in pig feces [18] is consistent with the results of the current study although the primary genera in pig feces at 10 weeks of age were *Prevotella* and *Lactobacillus* (25 and 11%, respectively) compared to *Clostridium*, *Lactobacillus*, and *Streptococcus* in the ileal mucosa at week 8 post-weaning of the current study (57, 11, and 10%, respectively). In our initial assessment [5], the HIGHA+ was considered the standard diet and the diet against which all other dietary treatments were compared. Based on the percent similarity, the bacterial profiles were more similar between week 2 and 4 post-weaning in the digesta than in the mucosa but more similar between week 4 and 8 post-weaning in the mucosa than in the digesta from which we concluded that the core microbiota is more likely from the mucosa bacteria than the digesta bacteria. Sequencing of the ileal mucosa bacteria supported this conclusion where 44% (i.e. eleven out of twenty-five) of the bacterial species with >1% of the total sequences each did not change from week 2 to 8 post-weaning indicating a rapid development of a relatively stable core microbiota. The change in bacterial populations over time in the current study are consistent with our initial assessment and those previously reported but the changes over time must be regarded with caution because time is confounded with differences in diet during the starter phase. Similarly, changes in total bacteria numbers were not measured in this study so results must be interpreted with caution.

Although there were minor statistical differences in bacterial composition due to diet complexity, the closer clustering at week 2 in pigs fed HIGH indicates a more uniform population compared to pigs fed LOW. The lack of bacterial population uniformity in LOW-fed pigs at week 2 may reflect differences in diet complexity, particularly given the lack of separation between HIGH and LOW-fed pigs at week 8. Further, the observed differences at week 2 were similar to that reported by others [21], [23], [24]. Differences due to diet complexity must be evaluated within the context that decreasing diet complexity was accomplished by altering inclusion of the major ingredients (i.e. cereals, fishmeal, whey), rather than a single ingredient. Simultaneously, changes in ingredient composition would alter composition of potential “functional” nutrients, nutrients that may provide a benefit beyond basic nutrition, including fiber concentration and type, polyunsaturated fatty acid composition and lactose content. Based on diet formulations the HIGH diet would contain higher levels of soluble fiber (i.e. β-glucan), polyunsaturated fatty acids and lactose relative to the LOW diet. Much of the microbiota data evaluating the interaction with functional nutrients are based on alterations in the fecal or luminal microbiome thus direct application to mucosal bacteria is unknown. Further, Longland et al [25] demonstrated that digestibility and utilization of energy from 2 different types of non-starch polysaccharide (NSP) fed to pigs were dependent on the basal diet composition such that energy digestibility and utilization was greater for each NSP type when included as the sole source of NSP than when included with another source of NSP. This may indicate an alteration in microbial populations associated with the combined ingredients relative to a single ingredient because in the pig microbial degradation is required for digestibility of energy supplied by NSP. Thus comparison of the current data with those from single ingredient, or single functional nutrient, studies must be conducted with caution; however, results observed in the current study are consist with observations based on changes in primary ingredients and hence functional nutrients.

In the current study, the LOW diet contained primarily corn and soybean meal, no barley, and minimal whey while the HIGH diet contained lower levels of corn and soybean meal with substantial contributions from barley, whey, and fishmeal. The LOW diet had higher proportions of Clostridiales and lower *Lactobacillus* and *Streptococcus* which is in accordance with that previously reported where diets based primarily on corn have lower *Lactobacillus* and *Streptococcus* and higher Clostridiales than diets based on barley [23], [24]. Relative abundance of *Streptococcus* in the ileal mucosa bacterial population was numerically lower in pigs fed a corn-based diet compared to a wheat/barley-based diet [25]. Further, differences in intestinal microbial profiles among cereal grains were correlated with differences in fiber composition [23]. The higher *Lactobacillus* populations in pigs fed the HIGH diet are in accordance with greater levels of whey and fishmeal in the diet [26], [27].

The lack of growth response to antibiotic at week 2 in pigs selected for microbial sequencing may have been due to the small sample size because there was a clear effect of antibiotic on pig growth based on the large performance trial [4]. Similar to diet complexity there were minor differences in mucosal bacterial profiles due to antibiotic inclusion at week 2; however, the lower microbial diversity at week 2 relative to week 8 is consistent with the expected effect of antibiotic inclusion [28]. This study utilized a single antibiotic thus the results may be somewhat dependent on the antibiotic used; however, Collier et al [28] reported no differences in similarity index between groups of pigs fed different antibiotic regimens but both antibiotic-treated groups were different than the untreated group. The increase in *Escherichia* with in-feed antibiotics at week 2 post-weaning is supported by Looft et al [21] where fecal abundance of the phylum Proteobacteria increased from 1–11% in medicated (APS250) pigs compared to non-medicated pigs and *Escherichia coli* made up 62% of the Proteobacteria in medicated pigs.

One of the objectives of the current study was to describe the changes in ileal mucosal bacterial profiles during periods of reduced and improved growth at week 2 and 8 post-weaning, respectively. At week 2 post-weaning, the proportion of *Clostridium leptum* was 2.5x greater in pigs fed LOW than HIGH but five times lower in pigs fed LOW than HIGH at week 8. *Clostridium leptum* is a proteolytic bacterial group which ferments undigested carbohydrate to produce butyrate [27]. The shift in abundance of *Clostridium leptum* from week 2 to 8 suggests a reduced diet digestibility at week 2, which is supported by lower growth performance and an improved efficiency of digestibility at week 8. The proportion of *Clostridium paraputrificum* was three times lower in pigs fed LOW than HIGH at week 2 but two times greater in pigs fed LOW than HIGH at week 8. *Clostridium paraputrificum* may confer some protection through degradation of the prebiotic chitin to chitosan [29] which exhibits antibacterial activities [30]. Furthermore, the *Sarcina* genus, associated with gastric ulcers in humans, dogs, and horses [31], [32] and abomasal bloat in ruminants [33], [34], declined from week 2 to 8 post-weaning in pigs fed LOW but increased with week post-weaning in pigs fed HIGH. The decrease in *Sarcina* and increase in *Clostridium paraputrificum* at week 8 post-weaning in pigs fed the low complexity diets in the weaning phase may have, in part, contributed to the improved growth observed at week 8 post-weaning in this same group of pigs. The positive correlation between *Clostridium paraputrificum* and pig body weight at week 2 and pig performance suggests a relationship between mucosal bacterial populations and diet complexity.

Another objective of the current study was to determine whether early dietary challenge as a result of reduced diet complexity or antibiotic exclusion may permanently alter the ileal mucosa bacterial profile. Thompson, et al [22] determined that there existed a ‘developmental window’ in which fecal microbial colonization was influenced by the immediate environment and may be more amenable to engineering or susceptible to disturbance. Provision of the experimental diets in the current study began at weaning (i.e. 21 d of age), during the ‘developmental window’, which was determined to be prior to 30 d of age when the fecal bacterial profile showed high stability (94.5% similarity between 30 and 36 d of age). In our initial assessment [5], the mucosa bacterial profile between the HIGHA+ and either the LOWA− or LOWA+ were less similar than between the HIGHA+ and HIGHA− diets at week 8 post-weaning indicating a prolonged effect of early diet complexity on the core microbiota. Based on the sequence data presented herein, however, the prolonged impact of early post-weaning nutrition on the ileal mucosa bacteria was variable. For example, the week post-weaning x antibiotic inclusion interaction indicated that early post-weaning nutrition influences prolonged composition of the genus *Sarcina*. The *Sarcina* genus were greater at week 2 post-weaning in pigs fed A+ compared to A− and remained greater at week 8 post-weaning despite the lack of in-feed antibiotics. However, for other species, the interaction between week post-weaning and diet complexity or antibiotic inclusion showed that differences which existed at week 2 were no longer different at week 8 post-weaning (i.e. *Escherichia albertii*, *Lactobacillus salivarus*, *Veillonella parvula*, *Clostridium sordellii*, *Escherichia fergusonii*) indicating that they were sensitive to changes in diet composition but that early post-weaning nutrition did not permanently alter colonization of these species. Furthermore, the lack of change in 44% of the bacterial species indicated that these species colonize early in the ileal mucosa and remain relatively stable over time despite changes in diet composition.

## Conclusions

Under different environmental conditions a given bacterial species may be classified as commensal or beneficial or possibly even pathogenic, thus unequivocal conclusions relating the changes in ileal mucosal bacteria observed in the current study to periods of reduced or improved growth cannot be made. However, given the stability of the mucosal bacteria over time observed in the current study, relative shifts in mucosal bacteria may play a role in pig performance during periods of reduced and improved growth. Further, early dietary challenge resulted in prolonged alterations in the mucosa bacterial profile but the effect was species specific and more associated with antibiotic inclusion than diet complexity which may have implications for evaluation of nutritional alternatives to in-feed antibiotics. This project is a unique assessment of microbial populations within the context of multi-ingredient (i.e. practical swine) diets and attempts were made to address possible confounding factors and maintain conclusions within the limitations of the study design.

## Supporting Information

Table S1 ^1^Bacterial genera with sequences of <1% of total reads were not included for statistical analysis. Pigs were fed diets differing in diet complexity and antibiotic inclusion from weaning (21 d of age) to 63 d of age (week 6 post-weaning). All pigs received the same grower-finisher diets from 64 to 78 d of age. At week 2 post-weaning *n* = 4, 5, 5, and 4 for HighA−, HighA+, LowA−, and LowA+, respectively. At week 8 post-weaning *n* = 6 for all treatment groups. Means values with their pooled standard errors. ^2^A, antibiotic inclusion; Comp, diet complexity. Within main effect of diet complexity or antibiotic inclusion, means within a row without common superscript differ ^a,b^ *P*<0.05 (Tukey’s means separation test).
(DOCX)

Table S2 ^1^Pigs were fed diets differing in diet complexity and antibiotic inclusion from weaning (21 d of age) to 63 d of age (week 6 post-weaning). All pigs received the same grower-finisher diets from 64 to 78 d of age. At week 2 post-weaning *n* = 4, 5, 5, and 4 for HighA−, HighA+, LowA−, and LowA+, respectively. At week 8 post-weaning *n* = 6 for all treatment groups. Means values with their pooled standard errors.
(DOCX)
