# Supplementary material for: Alterations in Ileal Mucosa Bacteria Related to Diet Complexity and Growth Performance in Young Pigs
Source: PLoS One. 2014 Sep 23;9(9):e108472. doi: 10.1371/journal.pone.0108472 (PMC4172762; doi:10.1371/journal.pone.0108472)
Supplement: Table S1 — 1Bacterial genera with sequences of <1% of total reads were not included for statistical analysis. Pigs were fed diets differing in diet complexity and antibiotic inclusion from weaning (21 d of age) to 63 d of age (week 6 post-weaning). All pigs received the same grower-finisher diets from 64 to 78 d of age. At week 2 post-weaning n = 4, 5, 5, and 4 for HighA−, HighA+, LowA−, and LowA+, respectively. At week 8 post-weaning n = 6 for all treatment groups. Means values with their pooled standard errors. 2A, antibiotic inclusion; Comp, diet complexity. Within main effect of diet complexity or antibiotic inclusion, means within a row without common superscript differ a,b P<0.05 (Tukey’s means separation test). (DOCX) [file pone.0108472.s001.docx]

**Table S1.** Mucosa bacterial species (expressed as a percentage of sequences) on the ileal mucosa of pigs fed diets differing in diet complexity and antibiotic inclusion at week 2 and 8 post-weaning based on 16S rRNA gene sequencing^1^.

| Diet Complexity | HIGH | |  | LOW | |  | *P*-value^2^ | | |
| --- | --- | --- | --- | --- | --- | --- | --- | --- | --- |
| Species | A- | A+ |  | A- | A+ | SEM | Comp | A | Comp x A |
| **Week 2** |  |  |  |  |  |  |  |  |  |
| *Lactobacillus crispatus* | 35.3 | 14.1 |  | 3.98 | 23.3 | 10.9 | 0.35 | 0.93 | 0.12 |
| *Lactobacillus iners* | 11.7 | 6.18 |  | 29.9 | 4.19 | 9.32 | 0.40 | 0.10 | 0.24 |
| *Lactobacillus salivarius* | 2.36 | -0.41 |  | 0.12 | 0.05 | 0.38 | 0.07 | **0.01** | **0.01** |
| *Lactobacillus reuteri* | 5.63 | 3.03 |  | 8.49 | 4.75 | 2.83 | 0.44 | 0.30 | 0.84 |
| *Clostridium leptum* | 0.82 | 22.4 |  | 34.8 | 19.7 | 13.3 | 0.28 | 0.81 | 0.22 |
| *Clostridium butyricum* | 4.17 | 4.17 |  | 1.03 | 0.14 | 2.28 | 0.18 | 0.85 | 0.85 |
| *Clostridium sordellii* | 0.40 | 5.76 |  | 0.79 | 12.6 | 5.18 | 0.50 | 0.16 | 0.55 |
| *Clostridium difficile* | 0.10 | 0.79 |  | 0.18 | 0.77 | 0.38 | 0.93 | 0.15 | 0.90 |
| *Clostridium paraputrificum* | 1.14 | 5.35 |  | 1.37 | 0.18 | 3.05 | 0.44 | 0.63 | 0.40 |
| *Clostridium bifermentans* | 0.54 | 2.73 |  | 0.80 | 2.79 | 1.52 | 0.92 | 0.22 | 0.95 |
| *Clostridium hiranonis* | 0.19 | 1.48 |  | 0.23 | 1.77 | 0.88 | 0.85 | 0.16 | 0.89 |
| *Clostridium metallolevans* | 4.08 | 3.55 |  | 0.69 | 0.54 | 2.38 | 0.23 | 0.89 | 0.94 |
| *Clostridium glycolicum* | 1.16 | 0.55 |  | 0.15 | 0.04 | 0.49 | 0.18 | 0.49 | 0.62 |
| *Clostridium perfringens* | 2.10 | 1.02 |  | 2.74 | 0.01 | 1.43 | 0.90 | 0.21 | 0.55 |
| *Prevotella oris* | 0.14 | 0.03 |  | 0.46 | 0.15 | 0.15 | 0.18 | 0.18 | 0.45 |
| *Prevotella copri* | 0.54 | 0.35 |  | 2.15 | 0.72 | 0.81 | 0.26 | 0.33 | 0.44 |
| *Megasphaera elsdenii* | 0.36 | 0.16 |  | 0.69 | 0.43 | 0.35 | 0.42 | 0.49 | 0.92 |
| *Sarcina* (species unknown) | 2.27 | 5.71 |  | 4.19 | 13.2 | 5.73 | 0.43 | 0.29 | 0.61 |
| *Veillonella parvula* | 1.08 | 0.11 |  | 3.15 | 0.07 | 1.43 | 0.50 | 0.21 | 0.48 |
| *Escherichia albertii* | 1.96 | 2.53 |  | 0.27 | 0.57 | 1.11 | 0.16 | 0.70 | 0.90 |
| *Escherichia fergusonii* | 1.91 | 5.81 |  | 0.48 | 3.53 | 2.02 | 0.38 | 0.14 | 0.83 |
| *Selenomonas ruminantium* | 0.18 | 0.24 |  | 0.73 | 0.42 | 0.31 | 0.27 | 0.69 | 0.55 |
| *Turicibacter* (species unknown) | 3.64 | 3.19 |  | 0.23 | 1.33 | 2.09 | 0.26 | 0.88 | 0.72 |
| *Streptococcus hyointestinalis* | 2.94 | 1.96 |  | 0.14 | 0.34 | 1.34 | 0.16 | 0.78 | 0.67 |
| *Streptococcus alactolyticus* | 0.11 | 0.06 |  | 0.04 | 0.40 | 0.16 | 0.42 | 0.36 | 0.26 |
|  |  |  |  |  |  |  |  |  |  |
| **Week 8** |  |  |  |  |  |  |  |  |  |
| *Llactobacillus crispatus* | 1.49 | 0.93 |  | 0.67 | 5.10 | 1.48 | 0.29 | 0.23 | 0.13 |
| *Lactobacillus iners* | 12.8 | 2.41 |  | 1.03 | 6.14 | 5.49 | 0.49 | 0.65 | 0.20 |
| *Lactobacillus salivarius* | 0.00 | 0.01 |  | 0.03 | 0.02 | 0.02 | 0.44 | 0.82 | 0.51 |
| *Lactobacillus reuteri* | 2.95 | 2.25 |  | 0.58 | 7.70 | 2.44 | 0.55 | 0.22 | 0.15 |
| *Clostridium leptum* | 9.36 | 1.27 |  | 1.66 | 0.28 | 4.55 | 0.37 | 0.33 | 0.48 |
| *Clostridium butyricum* | 19.1 | 10.1 |  | 16.3 | 19.5 | 6.16 | 0.61 | 0.62 | 0.31 |
| *Clostridium sordellii* | 5.37 | 2.79 |  | 4.00 | 2.25 | 2.24 | 0.68 | 0.36 | 0.85 |
| *Clostridium difficile* | 1.41 | 0.56 |  | 1.29 | 0.52 | 0.56 | 0.89 | 0.18 | 0.95 |
| *Clostridium paraputrificum* | 6.60 | 6.43 |  | 18.08 | 8.61 | 3.17 | 0.06 | 0.17 | 0.18 |
| *Clostridium bifermentans* | 6.23 | 3.21 |  | 7.46 | 3.09 | 2.36 | 0.82 | 0.16 | 0.78 |
| *Clostridium hiranonis* | 1.41 | 0.97 |  | 2.65 | 0.61 | 0.90 | 0.63 | 0.20 | 0.40 |
| *Clostridium metallolevans* | 3.93 | 10.8 |  | 11.00 | 13.3 | 3.97 | 0.27 | 0.27 | 0.57 |
| *Clostridium glycolicum* | 0.78 | 1.90 |  | 2.39 | 2.50 | 0.77 | 0.19 | 0.43 | 0.52 |
| *Clostridium perfringens* | 0.88 | 6.23 |  | 0.17 | 0.94 | 3.00 | 0.35 | 0.31 | 0.45 |
| *Prevotella oris* | 0.05 | 0.03 |  | 0.03 | 0.06 | 0.03 | 0.94 | 0.96 | 0.52 |
| *Prevotella copri* | 1.77 | 0.30 |  | 0.41 | 0.68 | 0.71 | 0.51 | 0.42 | 0.26 |
| *Megasphaera elsdenii* | 0.09 | 0.13 |  | 0.18 | 0.40 | 0.20 | 0.39 | 0.54 | 0.64 |
| *Sarcina* (species unknown) | 1.29 | 17.0 |  | 2.75 | 5.04 | 2.90 | 0.11 | **0.01** | 0.05 |
| *Veillonella parvula* | 0.06 | 0.02 |  | 0.10 | 0.04 | 0.03 | 0.37 | 0.13 | 0.75 |
| *Escherichia albertii* | 0.03 | 0.16 |  | 0.04 | 0.06 | 0.06 | 0.43 | 0.20 | 0.36 |
| *Escherichia fergusonii* | 0.13 | 0.29 |  | 0.07 | 0.09 | 0.10 | 0.24 | 0.39 | 0.51 |
| *Selenomonas ruminantium* | 0.17 | 0.13 |  | 0.21 | 0.30 | 0.18 | 0.57 | 0.90 | 0.73 |
| *Turicibacter* (species unknown) | 10.4 | 2.10 |  | 8.73 | 4.56 | 3.15 | 0.90 | 0.08 | 0.53 |
| *Streptococcus hyointestinalis* | 0.04 | 0.56 |  | 1.52 | 0.27 | 0.86 | 0.51 | 0.67 | 0.32 |
| *Streptococcus alactolyticus* | 3.88 | 15.7 |  | 8.27 | 8.21 | 8.11 | 0.85 | 0.49 | 0.48 |
