# Supplementary material for: Alterations in Ileal Mucosa Bacteria Related to Diet Complexity and Growth Performance in Young Pigs
Source: PLoS One. 2014 Sep 23;9(9):e108472. doi: 10.1371/journal.pone.0108472 (PMC4172762; doi:10.1371/journal.pone.0108472)
Supplement: Table S2 — 1Pigs were fed diets differing in diet complexity and antibiotic inclusion from weaning (21 d of age) to 63 d of age (week 6 post-weaning). All pigs received the same grower-finisher diets from 64 to 78 d of age. At week 2 post-weaning n = 4, 5, 5, and 4 for HighA−, HighA+, LowA−, and LowA+, respectively. At week 8 post-weaning n = 6 for all treatment groups. Means values with their pooled standard errors. (DOCX) [file pone.0108472.s002.docx]

**Table S2.** Correlation coefficient at r value between pig performance at week 2 and 8 post-weaning and ileal mucosa bacterial species of pigs fed diets differing in complexity or antibiotic inclusion^1^.

| Species | Correlation coefficient | *P*-value |
| --- | --- | --- |
| Average daily gain, week 2 |  |  |
| *Clostridium Leptum* | -0.09 | 0.717 |
| *Clostridium paraputrificum* | 0.10 | 0.661 |
| *Sarcina* (species unknown) | -0.24 | 0.314 |
| Body weight, week 2 |  |  |
| *Clostridium Leptum* | -0.14 | 0.567 |
| *Clostridium paraputrificum* | 0.57 | **<0.01** |
| *Sarcina* (species unknown) | -0.24 | 0.308 |
| Average daily gain, week 8 |  |  |
| *Clostridium Leptum* | 0.40 | **0.050** |
| *Clostridium paraputrificum* | -0.10 | 0.641 |
| *Sarcina* (species unknown) | -0.05 | 0.802 |
| Body weight, week 8 |  |  |
| *Clostridium Leptum* | -0.07 | 0.758 |
| *Clostridium paraputrificum* | -0.06 | 0.765 |
| *Sarcina* (species unknown) | -0.12 | 0.581 |
